# Cardiovascular disease risk among Chinese antiretroviral-naïve adults with advanced HIV disease

**DOI:** 10.1186/s12879-017-2358-0

**Published:** 2017-04-20

**Authors:** Fuping Guo, Evelyn Hsieh, Wei Lv, Yang Han, Jing Xie, Yanling Li, Xiaojing Song, Taisheng Li

**Affiliations:** 1 Department of Infectious Diseases, Peking Union Medical College Hospital, Chinese Academy of Medical Sciences, Beijing, China; 2 Department of Internal Medicine, Yale School of Medicine, New Haven, CT USA

**Keywords:** HIV, Cardiovascular disease risk, Framingham risk score, Data collection on adverse events of anti-HIV drugs (D:A:D) risk score, Atherosclerotic cardiovascular disease risk score

## Abstract

**Background:**

Cardiovascular disease (CVD) is an important cause of mortality among HIV-infected patients, however little is known about the burden of CVD among this population in Asia. We sought to quantify prevalence of CVD risk factors, 10-year CVD risk, and patterns of CVD risk factor treatment in a group of individuals with HIV in China.

**Methods:**

We retrospectively analyzed baseline data from treatment-naïve HIV-infected adults enrolled in two multicenter clinical trials in China. Data regarding CVD risk factors such as smoking, hypertension, diabetes, dyslipidemia and obesity were assessed. The Framingham Risk Score (FRS) and Data Collection on Adverse Events of Anti-HIV Drugs (D:A:D) risk scores were calculated to estimate 10-year CVD risk. The American College of Cardiology/American Heart Association Atherosclerotic Cardiovascular Disease (ASCVD) Risk Score was used to identify individuals meeting criteria for lipid-lowering therapy.

**Results:**

In total, 973 patients were included in the analysis. Mean age was 36.0 ± 10.2 years and 74.2% were men. The most common CVD risk factors were dyslipidemia (51.7%) and smoking (23.7%). Prevalence of hypertension, diabetes and obesity were 8.4%, 4.6% and 1.0%, respectively. Over 65% of patients had at least one CVD risk factor. The prevalence of 10-year risk of CVD ≥10% was 4.5% based upon FRS and was 3.3% based upon D:A:D risk score. Few patients with dyslipidemia, hypertension or diabetes were on treatment.

**Conclusions:**

CVD risk factors are common but under-treated among Chinese treatment-naïve individuals with HIV. Future interventions should focus on training HIV providers to appropriately recognize and manage CVD risk factors during routine clinical assessments.

## Background

Increasing access to antiretroviral therapy (ART) has ostensibly changed HIV infection in many parts of the world from a fatal diagnosis to a chronic condition requiring lifelong monitoring and treatment. However, this extended life expectancy comes with unique long-term complications. Prior studies, largely from the USA and Europe, have demonstrated that cardiovascular disease (CVD) is a common cause of morbidity and mortality among individuals with HIV, and the second cause of non-AIDS related deaths (1.6 per 1000 person-years) after liver disease [1, 2]. Causes for the increased CVD risk observed among HIV-infected patients include both traditional risk factors, including aging, higher smoking rates, dyslipidemia, insulin resistance, deposition of body fat; and non-traditional factors, including inflammation, direct effects of HIV on the vasculature, and toxicity from ART [3, 4].

Several Western studies have demonstrated an increased risk of CVD among HIV-infected patients compared with HIV-negative populations using validated tools such as the Framingham CVD Risk Score (FRS), Data Collection on Adverse events of Anti-HIV Drugs (D:A:D) risk scores and the American College of Cardiology/American Heart Association (ACC/AHA) Atherosclerotic Cardiovascular Disease Risk Score (ASCVD) [4–8]. However, few studies have focused on CVD risk assessment among individuals with HIV in Asia. Two studies from Thailand found the prevalence of predicted cardiovascular risk in HIV-infected Thai patients was relatively low [9, 10]. In China, the prevalence of risk factors for CVD is high in the general population [11, 12], and CVD has become the leading cause of morbidity and mortality in recent decades [13]. No studies to date have assessed the underlying prevalence of CVD risk factors among Chinese individuals with HIV prior to initiation of ART.

To address this gap, we compiled baseline data from two large multicenter studies of individuals with HIV across China from 2009 to 2012, and measured the prevalence of CVD risk factors, 10-year CVD risk, and patterns of CVD risk factor treatment prior to initiation of ART.

## Methods

### Study design

We performed a retrospective cross-sectional analysis of baseline data collected as part of two large multicenter clinical trials from 2009 to 2012 across 11 provinces and municipalities of China that are regions of high HIV prevalence, including Beijing, Fujian, Guangdong, Guangxi, Henan, Hunan, Liaoning, Shanghai, Shanxi, Sichuan, and Yunnan province. The patients therefore represented a broad cross-section of the overall population of HIV-infected patients in China. The details of these clinical trials have been previously described [14, 15].

Data from all participants enrolled in the two multicenter clinical trials were considered for inclusion in this analysis. All participants were adults aged 18–65 years, with documented HIV infection confirmed by Western blot analyses, CD4+ cell count <500 *cells/mm* ^*3*^, and antiretroviral-naive. From a total of 1607 patients enrolled in the two parent studies, 973 patients (61%) had complete data regarding CVD risk factors and were included in the present analysis.

### Measurements

For each parent study, detailed sociodemographic and clinical data were collected by trained study staff at the baseline encounter and recorded in electronic research databases [13, 14]. Fasting plasma samples were collected as part of routine screening for HIV care at each study site, and results were subsequently entered into the electronic study database as part of the parent study protocols.

#### Sociodemographic and clinical characteristics

Data for the following sociodemographic and clinical variables were analyzed: age, sex, height, weight, body mass index (BMI), route of HIV transmission, years since HIV diagnosis, medical co-morbidities, and medication history. Data regarding smoking history was collected for each participant in the parent trials and included current smoking (yes/no) as well as frequency of smoking and number of cigarettes per day. BMI was further classified according to the guidelines established by the United States National Institutes of Health National Heart, Lung, and Blood Institute, with the following categorizations: underweight <18.5, normal 18.5–24.9, overweight 25–29.9, and obese ≥30.0 kg/m^2^ [16].

Blood pressure (mmHg) was measured using a manual sphygmomanometer on the patient’s arm while the patient was sitting at rest. Hypertension was defined based upon the Joint National Committee on Prevention, Detection, Evaluation and Treatment of High Blood pressure recommendations [17]. Patients with a systolic blood pressure (SBP) of at least 140 mmHg or diastolic blood pressure (DBP) of at least 90 mmHg on two or more visits, and patients prescribed antihypertensive medication were considered to have hypertension.

Dyslipidemia was defined as total cholesterol (TC) >5.2 mmol/l, high-density lipoprotein cholesterol (HDL-c) <1.0 mmol/l, low-density lipoprotein cholesterol (LDL-c) >4.1 mmol/l, or triglycerides (TG) >1.7 mmol/l based upon the United States National Cholesterol Education Program, Adult Treatment Panel (NCEP-ATP) III guidelines [18].

Patients were classified with diabetes if they had a prior diagnosis, a fasting plasma glucose ≥7.0 mmol/l or were being treated with insulin or oral hypoglycemic agents [10].

#### Laboratory analyses

For the purposes of this study we collected the following laboratory data from the two parent clinical trial databases: fasting plasma TC, HDL-c, TG, LDL-c, and glucose levels. As well plasma CD4+ cell counts and HIV-1 RNA viral load data were collected. The laboratory techniques used to obtain these measurements have been described previously [13, 14].

#### Cardiovascular disease risk classification

We employed three CVD risk prediction models including the FRS, D:A:D risk scores and the ACC/AHA ASCVD Risk Score. For individuals ≥20 years of age, the FRS calculates 10-year predicted risk of CVD based upon a model comprised of age, sex, TC, HDL-c, SBP, current treatment for hypertension and cigarette smoking (anytime in the past month) [18]. The CVD risk prediction model also categorizes individuals as low (<10% 10-year risk), intermediate (10–20% risk) or high risk (≥20% risk) for coronary heart disease [3, 19, 20].

D:A:D 10-year estimated CVD risk scores were calculated based upon age, gender, current smoking, ex-smoking status, total and HDL cholesterol, systolic blood pressure, diabetes, family history of CVD and prior use of specific ART [4, 21]. Patients in our study were ART-naïve at enrollment and had no known family history of CVD. Therefore, the value for covariates in the algorithm representing specific ART drugs conferring CVD risk (duration of indinavir, lopinavir, abacavir) were set to zero and no prior family history of CVD was assumed in the calculation of the D:A:D risk prediction equations as has been described previously [10].

For individuals ≥40 years of age, we also calculated the ACC/AHA ASCVD Risk Score based upon age, sex, race, total cholesterol, HDL-c, SBP, current treatment for hypertension, diabetes and current smoking (yes/no) (http://tools.acc.org/ASCVD-Risk-Estimator/). This score estimates a 10-year risk for ASCVD for patients aged 40 to 79 years.

We further estimated the proportion of individuals with a favorable CVD risk profile, which was defined on the basis of a number of modifiable risk factors as follows: total cholesterol <5.17 mmol/l, SBP ≤120 mmHg, DBP ≤80 mmHg, no current smoking, no diabetes and no prior CVD [10, 22].

According to the 2013 ACC/AHA guidelines, statin therapy should be recommended for patients if they meet the following criteria: age ≥ 21 years with LDL-c≥ 4.9 mmol/l, age 40–75 years with diabetes and LDL-c 1.8–4.9 mmol/l, or age 40–75 years with 10-year ASCVD risk score of 7.5% or higher [23]. We determined the number of patients in our cohort who should be on statin therapy according to these guidelines.

### Ethics

Written informed consent was obtained from all individual participants prior to enrollment in the parent trials. The two studies were reviewed and approved by the institutional review board of the Peking Union Medical College Hospital prior to initiation. All procedures performed were carried out in accordance with the ethical standards laid down in the 1964 *Declaration of Helsinki* and its later amendments.

### Statistical analysis

All statistical analyses were performed using the SPSS 19.0 statistical software package (IBM Corporation, Armonk, New York, USA). Descriptive data were tabulated and reported using simple means, standard deviations, medians, interquartile ranges, and frequencies. Stratified analyses based upon gender and age strata (20–29, 30–39, 40–49, 50–59, ≥60 years) were assessed using the Student’s *t*-test for parametric continuous variables, Wilcoxon Rank Sum test for non-parametric continuous variables, and the Chi-squared test for categorical variables. Univariate and multivariable logistic regression [24] were performed to assess the relationship between the dependent variables of interest (FRS ≥10%, D:A:D risk score ≥ 10%, and ASCVD risk score ≥ 10%) and the independent variables of interest [age, sex, current smoking, BMI, hypertension, diabetes, dyslipidemia, transmission route, baseline CD4+ cell count (categorized as <200, 200–349, and >350 cells/mm^3^), and baseline viral load]. Variables with *p* < 0.2 in the univariate analyses were included in multivariable regression. All tests performed were two-tailed, with *p* < 0.05 considered to be statistically significant.

## Results

### Sociodemographic and clinical characteristics

Table 1 summarizes the characteristics of the 973 Chinese adult ART-naïve patients whose data were included in the study. The patient population was primarily male (722/973, 74.2%). The mean age was 36.0 ± 10.2 years, ranging from 18 to 65 years. Mean CD4+ cell count was 229 ± 123 cells/mm^3^, and viral load was 4.69±0.70 log10 copies/ml. Compared to our sample, the overall population of 1607 patients from the two parent trials was older (38.0 ± 11.0 years, *p* < 0.001) and had a somewhat lower proportion of male participants [1132 (70.4%), *p* = 0.039], however did not differ significantly based upon other clinical or CVD risk characteristics.

**Table 1:** Demographic and clinical characteristics of study participants, overall and by sex

Characteristic	All (*n* = 973)	Male (*n* = 722)	Female (*n* = 251)	*p*-value
Age years	36.0 ± 10.2	35.1 ± 10.1	38.7 ± 10.1	<0.001
Route of transmission				<0.001^b^
MSM	382 (39.3%)	382 (52.9%)	0	
Heterosexual	466 (47.9%)	248 (34.3%)	218 (86.9%)	
Blood-borne	51 (5.2%)	32 (4.4%)	19 (7.6%)	
Other/unknown	74 (7.6%)	60 (8.3%)	14 (5.6%)	
Years since HIV diagnosis	1.0 ± 1.6	0.8 ± 1.3	1.6 ± 2.1	0.01
CD4+ count cells/mm^3^ (*N* = 969)	229 ± 123	227 ± 124	233 ± 121	0.52
HIV RNA log10 copies/ml (*N* = 710)	4.69 ± 0.70	4.74 ± 0.67	4.56 ± 0.77	<0.01
Current smoking	231 (23.7%)	223 (30.9%)	8 (3.2%)	<0.001
BMI kg/m^*2*^	21.6 ± 2.9	21.5 ± 2.7	21.6 ± 3.3	0.82
Overweight	94 (9.7%)	63 (8.7%)	31 (12.4%)	0.09
Obesity	10 (1.0%)	5 (0.7%)	5 (2.0%)	0.08
Systolic blood pressure mmHg	115 ± 13	116 ± 12	113 ± 14	<0.01
Diastolic blood pressure mmHg	75 ± 9	75 ± 9	75 ± 10	0.79
Hypertension	82 (8.4%)	56 (7.8%)	26 (10.4%)	0.20
Blood glucose mmol/l	5.3 ± 1.0	5.3 ± 1.0	5.3 ± 1.1	0.77
Diabetes	45 (4.6%)	33 (4.6%)	12 (4.8%)	0.89
TC mmol/l	4.1 ± 1.0	4.0 ± 0.9	4.2 ± 1.1	<0.01
TG mmol/l	1.5 ± 1.1	1.5 ± 1.1	1.5 ± 1.0	0.21
HDL-c mmol/l	1.2 ± 0.4	1.2 ± 0.4	1.3 ± 0.4	<0.001
LDL-c mmol/l	2.3 ± 0.8	2.3 ± 0.8	2.3 ± 0.9	0.95
TC >5.2 mmol/l	104 (10.7%)	66 (9.1%)	38 (15.1%)	<0.01
TG >1.7 mmol/l	282 (29.0%)	215 (29.8%)	67 (26.7%)	0.35
HDL-c < 1.0 mmol/l	299 (30.7%)	249 (34.5%)	50 (19.9%)	<0.001
LDL-c > 4.1 mmol/l	17 (1.7%)	9 (1.2%)	8 (3.2%)	0.04
Dyslipidemia	503 (51.7%)	388 (53.7%)	115 (45.8%)	0.03
Number of CVD risk factors^a^				
Only one	465 (47.8%)	361 (50.0%)	104 (41.4%)	0.019
Two or more	194 (19.9%)	164 (22.7%)	30 (12.0%)	<0.001

Values are *n* (%) or mean ± standard deviation

MSM, men who have sex with men; BMI, body mass index; TC, total cholesterol; TG, triglycerides; HDL-c, high-density lipoprotein cholesterol; LDL-c, low-density lipoprotein cholesterol; CVD, cardiovascular disease; NA, not applicable

^a^Composite of current smoking, obesity (BMI ≥ 30.0 kg/m^2^), hypertension, diabetes, or dyslipidemia

^b^Not including MSM category

The most common CVD risk factors in this cohort were dyslipidemia (51.7%) and current smoking (23.7%). The prevalence of hypertension, diabetes and obesity were 8.4%, 4.6% and 1.0%, respectively. The majority of patients (67.7%) had at least one CVD risk factor (current smoking, obesity, diabetes, hypertension or dyslipidemia), and 19.9% had two or more risk factors. Based upon the NCEP-ATP III guidelines, 10.7% of individuals in our cohort met criteria for elevated TC levels, 29.0% had elevated TG levels, 30.7% had low HDL-c levels, and 1.7% had elevated LDL-c levels (Table 1).

Compared with female patients (Table 1), male patients were younger, had a higher prevalence of smoking and dyslipidemia, and higher viral loads. Among those meeting criteria for dyslipidemia, women were more likely than men to have elevated TC and LDL-c thresholds, whereas men were more likely than women to have low HDL-c levels (*p* < 0.05). Finally, a greater proportion of male patients had at least one CVD risk factor (72.7%) compared with their female counterparts (53.4%).

### Cardiovascular disease risk

**Fig. 1:**
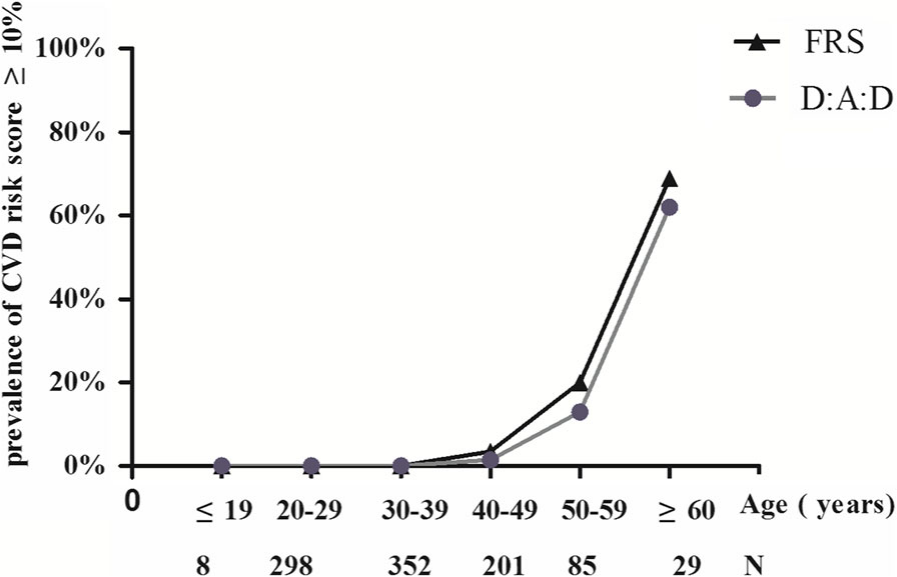
Prevalence of individuals with 10-year risk of CHD ≥ 10% according to the FRS and D:A:D Risk Score. FRS: the Framingham risk score; D:A:D: Data Collection on Adverse Events of Anti-HIV Drugs Risk Score

No patients had a known history of CVD at the time of enrollment. Table 2 shows the 10-year estimated CVD risk using the Framingham and D:A:D risk equations. Among 965 patients ≥20 years of age, 39 patients (4.0%) had a FRS between10 and 20%, and five individuals (0.5%) had a FRS ≥20%. Among 973 patients, 20 (2.1%) patients had a D:A:D risk score between10 and 20%, and 12 (1.2%) had a D:A:D risk score ≥ 20%. The proportion of patients with >10% estimated10-year risk of CVD were 4.5% using the FRS and 3.3% using D:A:D (*p* = 0.15). All patients with FRS ≥10% were male. As shown in Fig. 1, all patients with FRS or D:A:D risk scores ≥10% were older than 40 years of age, and there were no differences in the proportion of individuals with 10-year estimated CVD risk ≥10% across different age strata between the two risk scores.

**Table 2:** Estimated 10-year cardiovascular risk among Chinese antiretroviral-naïve HIV-infected patients

Characteristics	All *n*/*N* (%)	Male *n*/*N* (%)	Female *n*/*N* (%)	*p*-value
FRS				NA
< 10%	921/965 (95.5%)	671/715 (93.8%)	250/250 (100%)	
10–20%	39/965 (4.0%)	39/715 (5.5%)	0/250 (0%)	
> 20%	5/965 (0.5%)	5/715 (0.7%)	0/250 (0%)	
D:A:D Risk Score				NA
< 10%	941/973 (96.7%)	692/722 (95.8%)	249/251 (99.2%)	
10–20%	20/973 (2.1%)	18/722 (2.5%)	2/251 (0.8%)	
> 20%	12 /973 (1.2%)	12/722 (1.7%)	0/251 (0%)	
Favorable Cardiovascular Risk Profile	364/973 (37.4%)	257/722 (35.6%)	107/251 (42.6%)	0.047

NA, not applicable; FRS, the Framingham Risk Score; (D:A:D) Risk Score, Data Collection on Adverse Events of Anti-HIV Drugs (D:A:D) Risk Scores; Favorable cardiovascular risk profile was defined on the basis of a number of modifiable risk factors as follows: total cholesterol <5.17 mmol/l, systolic blood pressure ≤ 120 mmHg, diastolic blood pressure ≤ 80 mmHg, no current smoking, no diabetes and no prior CVD

In the multivariable logistic regression analysis (Table 3), while older age and smoking were positively associated with FRS ≥10% and ASCVD ≥10%, HIV-specific parameters such as route of transmission, baseline viral load and CD4+ T cell count were not independent risk factors for 10-year risk of CHD using any of the CVD risk prediction models. None of the predictors were found to be significantly associated with D:A:D Risk Score in the multivariable model (data not shown).

**Table 3:** Logistic regression analysis of 10-year CVD risk ≥10%, by FRS or ASCVD risk score

Variable	FRS ≥ 10%		ASCVD ≥ 10%	
	Crude OR (95% CI)	Adjusted OR^a^ (95% CI)	Crude OR (95% CI)	Adjusted OR^a^ (95% CI)
Age years	1.28 (1.21, 1.35) ^#^	1.42 (1.27, 1.59) ^#^	1.35 (1.23, 1.49) ^#^	1.80 (1.28, 2.53) ^$^
Female	NA	--	NA	--
Current smoking	6.88 (3.62, 13.09) ^#^	61.70 (11.35, 335.57) ^#^	11.63 (4.43, 30.56) ^#^	334.16 (8.25, 13,531.31) ^$^
BMI	1.07 (0.97, 1.18)	--	0.93 (0.79 1.09)	--
Hypertension	3.99 (1.94, 8.23) ^#^	2.26 (0.54, 9.39)	2.16 (0.84, 5.56) ^†^	2.82 (0.32, 25.29)
Diabetes	7.45 (3.41, 16.27) ^#^	1.33 (0.20, 8.85)	10.79 (4.18, 27.84) ^#^	10.58 (0.60, 185.97)
Dyslipidemia	2.33 (1.20, 4.51) ^+^	2.44 (0.67, 8.85)	1.36 (0.58, 3.18)	--
Route of transmission				
MSM	Reference	Reference	Reference	
Heterosexual	2.43 (1.17, 5.05) ^+^	0.24 (0.04, 1.31)	1.31 (0.46, 3.71)	--
Blood	1.49 (0.32, 7.00)	NA	0.75 (0.08,7.03)	--
Other/unknown	1.54 (0.41, 5.75)	0.17 (0.02, 1.96)	0.85 (0.15 4.73)	--
Years since HIV diagnosis	0.90 (0.71, 1.14)	--	0.53 (0.27, 1.05)	--
CD4+ count cells/ mm^*3*^				
< 200	1.71 (0.63, 4.64)	--	1.17 (0.31, 4.47)	--
200–349	1.50 (0.55, 4.08)	--	1.47 (0.39, 5.55)	--
> 350	Reference	Reference	Reference	Reference
Viral load log10 copies/ml	1.67 (1.02, 2.73) ^+^	0. 80 (0.38, 1.71)	1.84 (0.97, 3.49) ^†^	1.93 (0.54, 6.86)

^*^Factors with *p* < 0.2 in the univariate analyses were included in multivariable regression. OR: Odds ratio; CI: Confidence interval; BMI: body mass index; MSM: Men who have sex with men; FRS, the Framingham Risk Score; ASCVD, Atherosclerotic Cardiovascular Disease Risk Score; NA: not applicable

^†^ *p* < 0.2, ^+^ *p* < 0.05, ^$^ *p* < 0.01, ^#^ *p* < 0.001

**Fig. 2:**
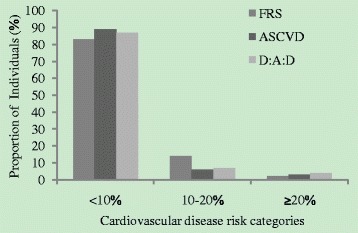
Cardiovascular disease (CVD) risk categories, by risk score. Ten-year CVD risk is depicted in three categories: <10%, 10–20% and >20%, for each of the three CVD risk prediction models. ASCVD: atherosclerotic cardiovascular disease risk score; FRS: the Framingham risk score; D:A:D: Data Collection on Adverse Events of Anti-HIV Drugs Risk Score

The ASCVD Risk Score was calculated in 247 of 307 patients who were ≥40 years of age. In the multivariable logistic regression analysis (Table 3), older age and smoking were positively associated with ASCVD ≥10%. As shown in Fig. 2, the proportions of patients with ≥10% estimated 10-year risk of CVD was 16.6% using the FRS, 12.1% using D:A:D risk estimators and 10.5% using the ASCVD Risk Score. In general, the D:A:D and ASCVD Risk Scores were not significantly different, however FRS Risk Scores were significantly higher than estimates derived from the other two prediction models (*p* = 0.01).

By contrast, 37.4% of patients had a favorable CVD risk profile, with women being more likely to have a favorable risk profile compared with men (*p* = 0.047) (Table 2).

### Treatment patterns for cardiovascular risk factors

Among the 973 patients in this cohort, 47(4.8%) patients (2 patients with LDL-c ≥4.91 mmol/l; 15 patients >40 years, with diabetes and LDL-c 1.81–4.91 mmol/l; 30 patients >40 years with ASCVD Risk Score ≥ 7.5%) met criteria for initiation of statin therapy according to the 2013 ACC/AHA guidelines. However, no patients in our study were currently being treated with statins.

Furthermore, only 5.6% (10/179) of patients with hypertension were currently receiving antihypertensive therapy, and only 4.6% (4/87) of patients meeting criteria for diabetes were currently receiving pharmacologic treatment for diabetes.

## Discussion

This is the first multicenter study in China, and one of the largest in Asia, to comprehensively assess CVD risk among adult ART-naïve HIV-infected patients using composite CVD risk scores, and to assess gaps in treatment of key modifiable CVD risk factors. While overall 10-year CVD risk based upon the FRS and D:A:D risk score was low, likely due to the young age of this cohort and comparatively low BMI, we nonetheless found that over 65% of individuals had at least one CVD risk factor, and that few patients with hypertension, diabetes or dyslipidemia were prescribed treatment.

Mortality from CVD has risen dramatically in China over the past two decades and is now the leading cause of death among Chinese men and women [13]. This is due to increasing prevalence of CVD risk factors (e.g. diabetes, hypertension, and dyslipidemia) driven by aging, dietary change, physical inactivity, increasing BMI, and strikingly high rates of smoking and second hand smoke exposure. Therefore, understanding baseline CVD risk among individuals with HIV in China, and ensuring early intervention for individuals with CVD risk factors is highly important.

For the purposes of this study, we were not able to recruit a comparator group of HIV-negative individuals with similar demographic and HIV risk characteristics. However, data from the general Chinese population provide us with a basis for drawing some comparisons. The China Noncommunicable Disease Surveillance Group carried out a study among a nationally representative sample of over 90,000 Chinese adults over 20 years of age, and found that the prevalence of smoking, overweight and obesity, hypertension, dyslipidemia, and diabetes were 58.16, 36.67, 30.09, 67.43, and 10.61%, respectively, among men; and 3.44, 29.77, 24.79, 63.98, and 8.73%, respectively, among women. [11]. Another recent study evaluating cardiovascular risk factors among a nationally representative sample of 23,010 Chinese adults aged 18 years and older found the prevalence of smoking, overweight and obesity, hypertension, dyslipidemia, and diabetes were 43.2, 37.2, 27.4, 55.2, and 5.0% respectively among men; and 2.9, 27.6, 21.6, 44.3, and 3.7% respectively among women [12]. Of note, the former study, which was published in 2012, used slightly different criteria to define these CVD risk factors, but the latter study, published in 2016, utilized the same definitions for CVD risk factors applied in our analysis. Among the treatment-naïve adults with HIV in our study, prevalence of hypertension and overweight/obesity were notably lower that that reported in the general population. This finding likely reflects the catabolic status among our participants in the setting of advanced HIV disease status, as evidenced by the low mean CD4+ T cell count of 229 ± 123 cells/mm^3^. However, interestingly, rates of dyslipidemia and diabetes were similar compared to the general population despite the lower mean BMI of this population, perhaps signifying altered lipid and glucose metabolism in the setting of untreated HIV infection. The prevalence of current smoking among patients in our study was lower than found in the two general population studies [11, 12]. While smoking data was systematically collected for all participants, we cannot exclude the possibility of underreporting of this risk factor.

In terms of other cardiovascular endpoints, our group has previously published echocardiographic data collected in a subgroup of participants from the China AIDS Clinical Trial 0810 [25]. At baseline, prior to ART initiation, the prevalence of echocardiographic abnormalities, including left ventricular systolic dysfunction and diastolic dysfunction, was significantly higher among 325 ART-naive persons living with HIV compared with 97 age- and sex-matched healthy controls, underscoring again the risk for CVD among individuals with HIV.

The Strategic Timing of Anti-Retroviral Treatment (START) trial, which recruited ART-naïve patients from six continents, found that the proportion of individuals having at least one CVD risk factor varied widely by geographic region (North America:70%; Europe/Australia/Israel: 65.1%; South America:49.4%; Asia: 37%; and Africa: 55.7%) [10]. Compared to the Asian cohort in the START trial, composed of 154 individuals with HIV from Thailand, participants in our cohort were older [34(28–42) v. 30(24–37) years] and had a notably lower median CD4+ T cell count [235(141–314) v. 604(561–677) cells/mm^3^]. While prevalence of hypertension was similar, patients in our cohort had a higher prevalence of diabetes (4.6% v. 1.3%), dyslipidemia (51.7% v. 13.0%) and smoking (23.7% v. 16.9%), but lower prevalence of obesity (1% v. 3.2%).

It is important to note, however, that in the START trial, dyslipidemia was defined by LDL-c ≥ 4.1 mmol/l or use of cholesterol-lowering drugs, which differs from the definition used in our study. Adopting the definition used by the START trial, the prevalence of dyslipidemia in our population decreased to 1.7%, and the prevalence of at least one CVD risk factor decreased from 67.8% to 35%. However, in our cohort, the majority of dyslipidemia was attributable to high TG and low HDL, which is consistent with previous published studies in low- and middle-income countries, including a recent study by Shen et al. focused on prevalence of dyslipidemia among Chinese ART-naïve patients in China [26–30]. This, combined with the significant rate of under-treatment of dyslipidemia in our population, led us to utilize the more comprehensive definition put forth by the NCEP-ATP III Guidelines.

In addition to CVD risk factor prevalence data, our study provides novel information regarding 10-year CVD risk among patients in our cohort. We chose to use the FRS because it has been used widely both internationally and in China to predict CVD risk among HIV-negative patients [31], and FRS has good predictive accuracy for subclinical carotid atherosclerosis [32]. The D:A:D risk equations were specifically constructed for use in HIV-infected populations [4]. Given the younger age of populations with HIV in China (68.4% of patients in our cohort were less than 40 years), the FRS and D:A:D risk equations are more widely applicable compared with tools such as the ACC/AHA ASCVD Risk Score, which applies to individuals ≥40 years of age. The prevalence of patients with 10-year CVD risk ≥10% in our cohort was 4.5% based upon the FRS, which is on par with that reported previously among other studies among Asian (6.5%) and African HIV-positive patients (3.6%) [10, 33] and lower compared with rates observed among individuals with HIV in Western countries (19.6–21.1%) [10]. The lower CVD risk in Asia and Africa in the START trial may reflect younger age and lower hypertension and obesity rates for the former, and higher proportion of women and low smoking and dyslipidemia rates in the latter. Consistent with these findings, patients in our study had lower rates of hypertension and obesity, but also more advanced HIV disease contributing to lower overall risk for CVD risk.

Among patients ≥40 years of age, lower overall CVD risk was attributed to the ART-naïve HIV-infected patients using the ASCVD model and the D:A:D risk score compared with the FRS. Prior studies have also suggested that the FRS overestimates CVD risk among individuals with HIV [34], however other studies have raised concerns about underestimation of CVD risk among individuals with HIV using the FRS as well [11]. In our cohort, the FRS and ASCVD Risk Scores both identified age and smoking as correlates of 10-year CVD risk ≥10%, which is not surprising given they are factored into the risk models themselves. However after adjusting for traditional risk factors no associations were observed between HIV-related factors and 10-year CVD risk ≥10%, irrespective of the risk algorithm used, similar to previous findings [35]. The D:A:D CVD Risk Score was developed and validated in a cohort of patients with HIV for the prediction of 5-year CVD risk, however the cohort used to develop the tool was largely European and American, and all patients were on ART. While it has been applied previously to study 10-year CVD risk in both treated and treatment-naïve patients with HIV [9, 10], the direct applicability of this algorithm to a relatively young treatment-naïve population in a low- and middle-income setting still requires formal validation, and findings should be interpreted with this caveat in mind.

Finally, prior studies from Western countries have highlighted the problem of underdiagnosis and undertreatment of CVD risk factors among ART-naïve HIV-infected patients [33]. One study from Italy demonstrated approximately 50% patients with HIV meeting criteria for statin therapy were not being treated [36]. Another study from France found HIV-infected patients treated with statins after acute coronary syndrome had less of an improvement in lipid profiles when compared with HIV-negative controls, in the setting of less potent statins and potential drug-drug interactions with antiretroviral drugs [37]. De Socio et al. found that one- to two-thirds of hypertensive HIV-infected patients in their study were unaware of their condition, and were not on antihypertensive therapy [38]. Furthermore, Zanni et al. found that compared with 2004 ATP III guidelines, under the 2013 ACC/AHA guidelines a higher percentage of patients met criteria for statin therapy, however, when patients were evaluated with contrast-enhanced coronary computed tomography angiography, significant discordance was observed between those initiated on statins and those found to have high risk morphology (HRM) coronary plaques. In fact the authors found that 74% of those with subclinical HRM coronary plaque would not be recommended statin therapy based upon the 2013 ACC/AHA guidelines alone [39]. Therefore, in addition to addressing underdiagnosis and undertreatment of CVD risk factors among individuals with HIV, future studies are also needed to evaluate the appropriateness of applying treatment guidelines developed for the general population to this population.

Few studies, however, have addressed this issue in resource-limited settings. In our cohort, we found significant under-treatment of dyslipidemia, hypertension and diabetes. Approximately 68% of individuals in our study had at least one risk factor for CVD. National clinical guidelines for HIV/AIDS care dictate that blood pressure is measured, and fasting lipid profile and glucose are checked prior to initiation of ART. However, care providers may be less well versed in the nuances of actual management of these primary care issues, particularly thresholds for initiating lipid lowering therapy. Furthermore, smoking cessation should be a cornerstone of primary CVD prevention, but is inadequately addressed in most HIV care settings in China. Further research is necessary to identify the prevention strategies that are most feasible and effective for reducing CVD risk within our population, and should combine both life-style modification strategies with pharmacologic management of hypertension, diabetes, and dyslipidemia [5, 14]. Although the currently existing CVD risk scores may not have been designed with our population in mind, they nevertheless provide a starting point for care providers to evaluate key CVD risk factors and identify individuals at highest-risk who may need close evaluation and management.

Our study has several limitations. First, our data are cross-sectional and therefore do not provide information regarding longitudinal change in CVD risk over time after initiation of ART, or regarding CVD events. Second, patients with CD4+ cell count ≥500 cells/mm^3^ were not enrolled in our study, which may explain why we did not see an association between CD4+ cell count and CVD risk. However, lack of correlation between CD4+ cell count and CVD risk has also been reported by Sabin et al. [40]. Third, the original studies from which these data are drawn were not designed with CVD risk assessment as their primary endpoints, therefore our analysis focuses on the subset of 973 patients who had complete CVD risk factor data available, which may influence the generalizability of our results. However, there is no known systematic bias influencing collection of risk factor data, and risk factor prevalence in the overall group was similar to that of the subset analyzed. Finally, in our study we did not have data on actual CVD clinical outcomes such as stroke or ischemic heart disease which would have enabled us to assess the ability of the FRS and ASCVD Risk Score to predict CVD in our cohort.

## Conclusions

In conclusion, although baseline predicted 10-year risk of CVD was low in this relatively young population, we found that CVD risk factors were common and significantly under-treated. More research is needed to understand the unique biology and epidemiology of HIV-associated CVD in Asia, to identify the optimal tools to assess CVD risk among individuals with HIV in Asia, and to assess the impact of prolonged infection and exposure to ART on CVD outcomes in this population. Finally, future interventions should focus on training HIV providers in China to appropriately recognize and manage CVD risk factors during routine clinical assessments.

## Abbreviations

ACC/AHA: American College of Cardiology/American Heart Association
ART: Antiretroviral therapy
ASCVD: Atherosclerotic Cardiovascular Disease
BMI: Body mass index
CVD: Cardiovascular disease
D:A:D: Data Collection on Adverse Events of Anti-HIV Drugs
DBP: Diastolic blood pressure
FRS: Framingham Risk Score
HDL-c: High-density lipoprotein cholesterol
HRM: High risk morphology
LDL-c: Low-density lipoprotein cholesterol
NCEP-ATP: National Cholesterol Education Program, Adult Treatment Panel
SBP: Systolic blood pressure
TC: Total cholesterol
TG: Triglycerides
